# Clinical and pathological analysis of pulmonary epithelioid vascular endothelial tumor

**DOI:** 10.1515/biol-2025-1259

**Published:** 2026-02-12

**Authors:** Dong-Yue Wang, Li-Bo Wang, Min Zhang, Jing Wu

**Affiliations:** Department of Pathology, Jilin Municipal People’s Hospital, Jilin, China; Department of Pathology, Affiliated Hospital of Inner Mongolia Medical University, Inner Mongolia, China

**Keywords:** hemangioendothelioma, epithelioid, lung neoplasms, treatment, prognosis

## Abstract

Pulmonary epithelioid hemangioendothelioma (PEHE) is a rare, low-grade malignant vascular tumor with nonspecific clinical and imaging features. Diagnosis remains challenging, particularly in intraoperative frozen sections and biopsy specimens, and relies on characteristic histopathological morphology and immunohistochemical findings, supplemented by molecular genetic testing when necessary. We report two cases of PEHE: a 72-year-old asymptomatic male with multiple pulmonary nodules detected incidentally on CT, initially diagnosed as “malignant tumor, likely metastatic” by frozen section during wedge resection but later confirmed as PEHE on paraffin pathology; and a 53-year-old female presenting with cough, sputum, dyspnea, and chest pain, whose CT showed scattered ground-glass and solid nodules, initially misdiagnosed as non-small cell carcinoma with nodal metastasis via percutaneous biopsy but ultimately confirmed as PEHE through immunohistochemistry (positive CD34/CD31/ERG). These cases underscore the diagnostic pitfalls of PEHE and aim to enhance awareness among clinicians and pathologists. A comprehensive review of current literature is also provided.

## 1 Background

Epithelioid hemangioendothelioma (EHE) is a rare, low-grade malignant vascular tumor with metastatic potential. The global incidence was 0.038/100,000, and the 5-year overall survival (OS) rate was 78.8 % [1]. Its annual incidence is <1 per million, representing <1 % of all vascular tumors [2]. In cases of single organ involvement, the most common site is the liver (∼34 %), followed by the skeleton (∼21 %) and the lungs (∼19 %) [3]. While EHE can arise in diverse anatomical sites – including soft tissues (limbs), bones, liver and lungs – pulmonary EHE (P-EHE) is exceptionally uncommon compared to other primary lung malignancies [2]. The diagnosis of P-EHE remains challenging due to its nonspecific clinical presentation and imaging features. This often leads to misdiagnosis during intraoperative frozen section analysis or needle biopsy. Definitive diagnosis requires integration of histopathological morphology, immunohistochemical staining (e.g., positivity for vascular markers such as CD31, CD34 and ETS-Related Gene (ERG)) and in equivocal cases, molecular genetic testing (e.g., WWTR1-CAMTA1 or YAP1-TFE3 fusions) [4], 5]. To improve diagnostic accuracy and clinical awareness,we analyzed the clinical data, pathological features, treatment strategies and follow-up outcomes of two P-EHE cases, supplemented by a comprehensive literature review. Our findings aim to assist clinicians and pathologists in recognizing this elusive tumor and refining diagnostic approaches.

### 1.1 Case 1

#### 1.1.1 Timeline

– August 2024: A chest computed tomography (CT) scan during a physical examination revealed multiple nodules in both lungs.
– September 6, 2024: Hospital admission and performance of “wedge resection of the left upper and left lower lung.” Intraoperative frozen section indicated malignancy.
– Mid-September 2024: Paraffin pathology and immunohistochemistry confirmed the diagnosis as pulmonary epithelioid hemangioendothelioma.
– December 2024: Three-month postoperative follow-up showed no discomfort in the patient. A follow-up CT scan is planned for six months later.

**Figure 1:**
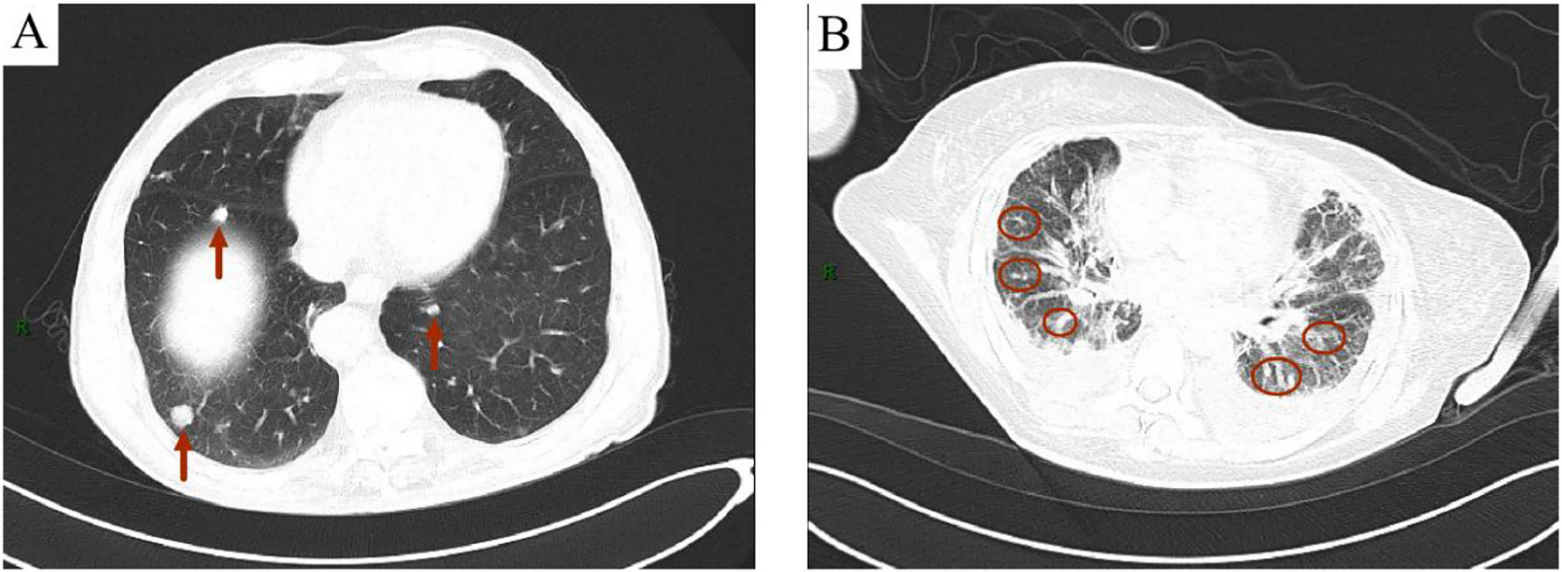
(Chest CT) (A) Case 1 multiple nodular and linear high-density shadows in both lungs (indicated by arrows); (B) Case 2 multiple patchy, linear, and nodular increased density shadows in both lungs, unclear boundaries (circled).

**Case descriptions:** A 72-year-old male was admitted following the incidental detection of bilateral pulmonary nodules on a physical examination one week prior. The patient was asymptomatic. Computed tomography (CT) revealed multiple pulmonary nodules, with the largest measuring ∼22.7 × 15.8 mm (Figure 1A). A wedge resection was performed, and intraoperative frozen section analysis reported “malignant tumor, likely metastatic.” Postoperative paraffin-embedded histopathology and immunohistochemical analysis (positive for CD34, CD31, and ERG; negative for cytokeratin (CK), thyroid transcription factor-1 (TTF-1), and a variant of P63 (P40)) confirmed the diagnosis of pulmonary epithelioid hemangioendothelioma.

**Figure 2:**
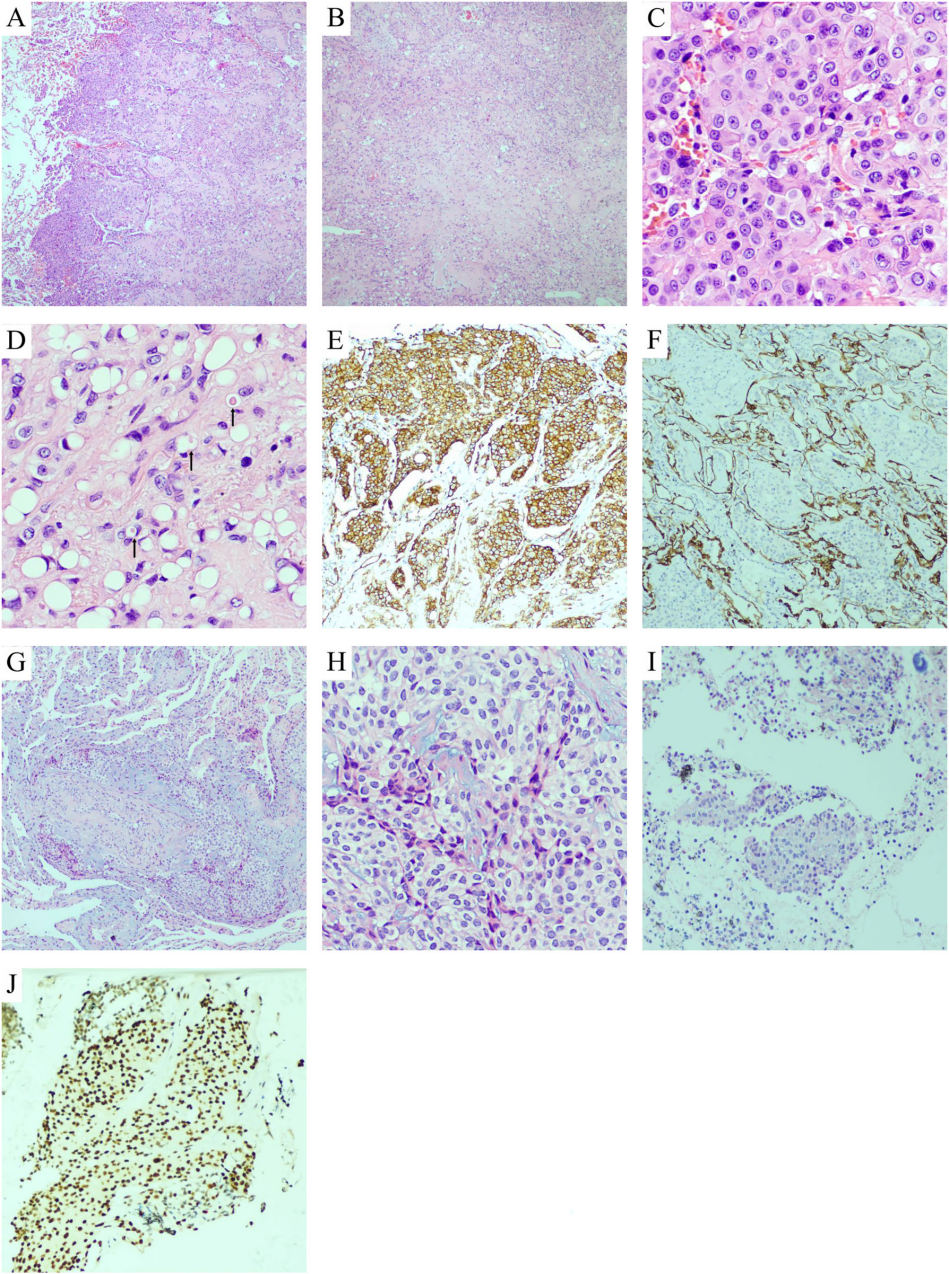
Case 1: (A) Multinodular tumor growth with dense peripheral tumor cells protruding into alveolar spaces (hematoxylin and eosin (stain) (HE), ×40). (B) Uneven tumor cell distribution: Hypocellular central area (collagen/myxoid background) and hypercellular periphery (HE, ×40). (C) Tumor cells in nests with epithelioid morphology, eosinophilic cytoplasm, and round-oval nuclei (HE, ×400). (D) Cord-like or scattered tumor cells with cytoplasmic vacuoles (arrows: RBC-containing vacuoles suggesting vascular lumen formation) (HE, ×400). (E) CD31 positivity on tumor cell membranes (IHC, ×100). (F) CK negativity highlights tumor cells within alveolar spaces (IHC, ×100). Case 2: (G) Lung biopsy: Tumor cells in nests/cords within myxoid stroma (HE, ×100). (H) Epithelioid tumor cells with eosinophilic cytoplasm and cytoplasmic vacuoles (HE, ×200). (I) Lymph node metastasis: Tumor nests with lymphoid background (HE, ×100). (J) Nuclear ERG expression in tumor cells (IHC, ×200).

**Histopathological findings**: Demonstrated well-circumscribed, multinodular tumors with hypocellular central hyalinized zones (Figure 2A) and peripheral cellular clusters protruding into alveolar spaces (Figure 2B). Tumor cells were arranged in nests/cords (Figures 2C and D), exhibiting epithelioid morphology with eosinophilic cytoplasm, round nuclei, and rare mitosis (Figure 2F). Intracytoplasmic vacuoles containing erythrocytes (primitive vasculogenesis) were noted (Figure 2E). Immunohistochemistry (IHC) showed positivity for CD34 (Figure 2G), CD31 (Figure 2H), ERG (Figure 2I), friend leukemia integration 1 (FLI-1), and vimentin, while being negative for epithelial (CK, Figure 2J), adipocytic (CDK4/MDM2), and neuroendocrine markers. Ki-67 index was 10 %.

### 1.2 Case 2

#### 1.2.1 Timeline

– September 2020: Underwent “thoracoscopic wedge resection of the right upper lung lobe” at an external hospital, with pathological diagnosis of pulmonary epithelioid hemangioendothelioma (specific details unknown).
– September 2022: Developed symptoms including cough, expectoration, shortness of breath, and chest pain.
– November 20, 2022: Hospital admission. CT scan revealed multiple pulmonary nodules with lymphadenopathy. Initial pathological diagnosis from biopsy suggested non-small cell carcinoma with lymph node metastasis.
– December 2022: Diagnosis revised to pulmonary epithelioid hemangioendothelioma with lymph node metastasis following immunohistochemical review.
– November 2024: Last follow-up. The patient remained in stable condition, with imaging reviews scheduled every six months.

**Case descriptions:** A 53-year-old female presented with a two-month history of cough, expectoration, shortness of breath, and chest pain. Computed tomography (CT) revealed multiple bilateral pulmonary nodules accompanied by lymphadenopathy (Figure 1B). Initial pathological diagnosis from biopsy suggested non-small cell carcinoma. However, upon further review of her medical history, it was discovered that she had been diagnosed with pulmonary epithelioid hemangioendothelioma in 2020. Additional immunohistochemical analysis, which showed positivity for CD34 and ERG and negativity for epithelial and neuroendocrine markers, led to a revision of the diagnosis to pulmonary epithelioid hemangioendothelioma with lymph node metastasis.

**Histopathological findings**: Biopsies revealed epithelioid tumor cells in myxoid stroma, with vacuolated cytoplasm. Lymph node sections showed similar tumor nests amid lymphocytes. IHC confirmed vascular marker (CD34, ERG) positivity and excluded carcinoma/neuroendocrine tumors.

### 1.3 Patient perspective

Both patients expressed concern regarding the rarity of the disease but felt reassured by the eventual definitive diagnosis. The patient in Case 1 was surprised by the asymptomatic discovery of the tumor and reported a calm mindset following surgical treatment. The patient in Case 2, having experienced an initial misdiagnosis, indicated feeling considerable anxiety during the diagnostic process and expressed a hope for enhanced multidisciplinary communication in the future to reduce diagnostic errors.

### 1.4 Follow-up plan

Case 1: Chest CT scan every six months to monitor for changes in the nodules.
Case 2: Chest CT scan and lymph node imaging every six months, with instructions to seek medical attention promptly if any new symptoms arise.

Both Cases: It is recommended that, should new lesions or symptoms appear, a multidisciplinary team consultation be considered to assess and plan subsequent treatment.

**Informed consent:** Informed consent has been obtained from all individuals included in this study.

**Ethical approval:** The research related to human use has been complied with all the relevant national regulations, institutional policies and in accordance with the tenets of the Helsinki Declaration, and has been approved by the authors’ Institutional Review Board or equivalent committee.

## 2 Discussion

Although the pathogenesis of PEH remains incompletely understood, several hypotheses have been proposed. The occurrence of EHE primarily involves two mechanisms. First, in terms of molecular genetics, the development of EHE requires the stimulation of angiogenesis in endothelial cells by monocyte chemoattractant protein-1 (MCP-1) to promote tumor progression. The WWTR1 and CAMTA genes are involved in tumor development and play significant roles in this process [4], 5]. Additionally, studies have identified a subgroup with YAP1-TFE3 gene fusion, which is also associated with the occurrence of EHE [4], 5]. The second mechanism suggests that EHE may be related to chronic Bartonella infection. After invading the human body, Bartonella bacilli can induce long-term infections in red blood cells and endothelial cells. Bartonella upregulates mitotic and pro-inflammatory genes, promotes vascular endothelial growth factor-mediated proliferation, induces cytoskeletal rearrangement, and inhibits endothelial cell apoptosis, indicating that chronic Bartonella infection may contribute to the development of EHE [4], 6]. Pulmonary epithelioid hemangioendothelioma (PEHE) is extremely rare among lung tumors [7]. It predominantly occurs in middle-aged women, with an incidence rate 2–4 times higher than in men. It can also affect children and the elderly, with reported cases ranging from 7 to 83 years of age, but it is most common around the age of 40 [8], 9]. Approximately half of the patients are asymptomatic [3], 4], 10], while those with symptoms primarily present with respiratory manifestations (e.g., cough, sputum production, dyspnea, hemoptysis) and chest pain [11]. In this report, one case involved a middle-aged woman with no symptoms, detected during a physical examination, while the other was an elderly man presenting with cough, sputum production, and chest pain. PEHE has the potential for metastasis. Local pleural metastasis occurs in ∼20.4 % of cases, lymph node metastasis in 10.8 %, and distant metastases to organs such as the liver, bones, and skin may also occur [10]. Sardaro et al. [12] reported a case of PEHE where multiple disseminated pleuropulmonary nodules were detected on thoracic examination. The patient underwent wedge resection of the lung, confirming the diagnosis of PEHE, and later developed metastases to the L4-L5 vertebrae and spleen. In this report, one patient developed lymph node metastasis two years after the initial diagnosis. Zhu et al. [10] reported one case of PEHE combined with lung adenocarcinoma and another case combined with pulmonary cryptococcosis. On imaging, CT typically reveals multiple bilateral pulmonary nodules, usually <20 mm in diameter. Smaller nodules often have regular shapes, while slightly larger nodules may exhibit shallow lobulation at the margins. Small nodules show no significant enhancement, whereas larger nodules or masses may exhibit mild delayed enhancement. On positron emission tomography/computed tomography (PET/CT) scans, lesions often demonstrate increased fluorodeoxyglucose (FDG) uptake, with a positive correlation between lesion size and maximum standardized uptake value (SUVmax) [8], 13]. In this report, both patients presented with multiple bilateral pulmonary nodules. In one case, the nodules were smaller than 20 mm with uncertain enhancement on contrast scans, while in the other case, the largest nodule measured 30 mm and was accompanied by hilar and mediastinal lymphadenopathy. The clinical manifestations and imaging features of PEHE are nonspecific, and definitive diagnosis relies on histopathological examination [14].

Histopathological examination of PEHE reveals a characteristic multinodular growth pattern with irregular cellular distribution. The tumor demonstrates hypocellular central areas with hyalinized sclerosis, while the periphery shows dense tumor cell proliferation within alveolar spaces. Tumor cells are arranged in nests and cords, exhibiting epithelioid morphology with mild atypia. A diagnostic feature is the presence of intracytoplasmic vacuoles containing red blood cells, representing primitive vascular lumen formation. Some cases may demonstrate osteoclast-like giant cells and osseous metaplasia [15], while the presence of spindle-shaped tumor cells or fibrinous pleuritis with tumor proliferation correlates with poorer prognosis [16]. Immunohistochemically, tumor cells consistently express vascular markers (CD34, CD31, ERG, FLI-1, and Factor VIII), though ∼30 % may show focal epithelial marker expression, potentially leading to misdiagnosis as carcinoma [10]. The Ki67 proliferation index is typically low (10 % in our cases), consistent with the tumor’s indolent behavior [9]. Definitive diagnosis requires characteristic histomorphology combined with positive vascular marker expression. Molecularly, 90 % of EHEs harbor WWTR1-CAMTA1 fusions (demonstrated by nuclear CAMTA1 expression) [17], while 10 % show YAP1-TFE3 fusions (with diffuse nuclear TFE3 positivity) [17]. These molecular subtypes exhibit distinct pathological features: WWTR1-CAMTA1 cases display classic morphology with irregular cell distribution, mild atypia, and hyalinized stroma, whereas YAP1-TFE3 cases show well-formed vasculature and prominent nucleoli [17], 18]. Zeng et al. [9] identified a novel germline PALB2 mutation alongside WWTR1-CAMTA1 fusion in one case. Our two cases showed typical features suggestive of WWTR1-CAMTA1 fusion, though molecular confirmation was not obtained due to financial constraints.

### 2.1 Differential diagnosis

Due to the rarity of P-EHE, it is prone to misdiagnosis and requires differentiation from several other conditions in clinical practice. Distinctions must be made from: 1. primary pulmonary tumors, such as adenocarcinoma and neuroendocrine tumors, based on cellular atypia and immunohistochemical markers (including TTF-1, NapsinA, neuroendocrine markers, and vascular origin markers); 2. metastatic tumors, such as metastatic liposarcoma (often positive for MDM2 and CDK4) or metastatic carcinoma (diffusely positive for epithelial markers), which can be distinguished by combining clinical history and immunohistochemistry; 3. pulmonary epithelioid angiosarcoma, which typically shows more significant atypia, frequent mitotic figures, and slit-like vascular spaces; 4. epithelioid sarcoma, characterized by loss of INI1 expression; and 5. sclerosing pneumocytoma, which features two distinctive cell types and four architectural patterns, with positivity for TTF1 and EMA.

In this report, Case 1 was clinically suspected to be multifocal lung cancer or metastatic carcinoma. It was misdiagnosed as malignant tumor (likely metastasis) during intraoperative frozen section analysis, but was ultimately confirmed by paraffin pathology and immunohistochemistry [19]. Case 2 was initially misdiagnosed as non-small cell carcinoma with lymph node metastasis; the diagnosis was later corrected after reviewing the clinical history and performing additional immunohistochemical tests. Both cases highlight that in the differential diagnosis of multiple pulmonary nodules, especially when intraoperative frozen sections or biopsy samples are limited, the possibility of P-EHE should be considered. Prompt application of vascular origin immunohistochemical markers can aid in achieving an accurate diagnosis.

Studies have found that surgical resection remains the primary treatment for P-EHE, with surgical removal being the optimal approach for unilateral solitary or multiple nodules [3], 14]. For patients whose conditions cannot be completely cured by surgery, those with postoperative recurrence, multi-organ involvement, or extensive metastases, postoperative radiotherapy and chemotherapy are often used as adjuvant treatments [5], 6], 11], 20]. The combination of carboplatin and paclitaxel is the most commonly employed chemotherapy regimen. In recent years, individualized therapeutic approaches – such as combining anti-angiogenic drugs (e.g., bevacizumab) with chemotherapy, or pairing them with mTOR inhibitors (e.g., rapamycin) and immune checkpoint inhibitors – have demonstrated certain efficacy in some cases [9], 12], 14].

Most cases of P-EHE have a favorable prognosis, with a median survival time of ∼4.6 years and a 5-year survival rate of around 60 % [21]. Common factors associated with a poor prognosis include: the presence of clinical symptoms (such as dyspnea, hemoptysis, or chest pain), weight loss, anemia, pleural effusion, pleural invasion, lymph node metastasis, distant metastasis, tumor diameter >3.0 cm, and the microscopic presence of spindle-shaped tumor cells. In this report, Case 1 was asymptomatic and is likely to have a better prognosis, whereas Case 2 has already developed lymph node metastasis and requires close follow-up [10], 22], 23].

## 3 Conclusions

Pulmonary epithelioid hemangioendothelioma is challenging to diagnose and is easily confused with lung cancer, metastatic tumors, and other conditions. A definitive diagnosis relies on pathological morphology and immunohistochemical analysis, supplemented by molecular testing when necessary. Treatment primarily involves surgical resection, while radiotherapy, chemotherapy, or targeted therapy may be used as adjuvant treatments for recurrent or metastatic cases. The prognosis is generally favorable, but patients with lymph node metastasis, pleural invasion, or significant symptoms tend to have poorer outcomes. Therefore, more case studies are needed to further enhance the understanding of this tumor among clinicians and pathologists.

## References

[1] Haughey AM, Moloney BM, O’Brien CM (2023). Epithelioid haemangioendothelioma: not simply a hepatic pathology. Clin Imag.

[2] Shiba S, Imaoka H, Shioji K, Suto T, Kuze T, Abe T (2018). Clinical characteristics of Japanese patients with epithelioid hemangioendothelioma: a multicenter retrospective study. BMC Cancer.

[3] Oda N, Maeda Y, Kiura K, Miyahara N (2021). Pulmonary epithelioid haemangioendothelioma mimicking lung cancer. BMJ Case Rep.

[4] Chen X, Wang Y, Che G, Shen C (2023). An extremely rare case of pulmonary epithelioid hemangioendothelioma. Thorac Cancer.

[5] Mesquita RD, Sousa M, Trinidad C, Pinto E, Badiola IA (2017). New insights about pulmonary epithelioid hemangioendothelioma: review of the literature and two case reports. Case Rep Radiol.

[6] Mao X, Liang Z, Chibhabha F, Li H, Liu M, Yang Y (2017). Clinico‐radiological features and next generation sequencing of pulmonary epithelioid hemangioendothelioma: a case report and review of literature. Thorac Cancer.

[7] Chen FX, Li JQ, Xie PK (2023). Pulmonary epithelioid hemangioendothelioma: a case of computed-tomography diagnostic analysis and follow-up. Asian J Surg.

[8] Liu H, Wang J, Lang J, Zhang X (2021). Pulmonary epithelioid hemangioendothelioma: imaging and clinical features. J Comput Assist Tomogr.

[9] Zeng H, Tang X, Tian X, Liu Y, Tian P (2023). Poor response to sintilimab plus chemotherapy in a pulmonary epithelioid hemangioendothelioma patient: a case report. Immunotherapy.

[10] Zhu JX, Xie Q, Zhong AH, Lin QH, Lan CQ (2021). Clinical analysis of 16 cases of pulmonary epithelioid hemangioendothelioma. Zhonghua Jiehe He Huxi Zazhi.

[11] Lin H, Cheng Y, Zhang C (2019). Research progress of pulmonary epithelioid hemangioendothelioma. Zhongguo Fei Ai Za Zhi.

[12] Sardaro A, Bardoscia L, Petruzzelli MF, Portaluri M, D’Errico MP, Rubini G (2014). Pulmonary epithelioid hemangioendothelioma presenting with vertebral metastases: a case report. J Med Case Rep.

[13] Chen S, Zhuang D, Jin H, Hu G (2024). Imaging manifestations of epithelioid endothelial tumors occurring simultaneously in the liver and both lungs and literature review. Magn Reson Imaging.

[14] Gong J, Tian F, Wang Q, Liu Z, Zhang X, Han X (2022). Case report: rare epithelioid hemangioendothelioma occurs in both main bronchus and lung. Front Med.

[15] Adamane SA, Deodhar KK, Gupta AM, Karimundackal G, Desai SB (2016). Pulmonary hemangioendothelioma with osteoclast-like giant cells: a rare observation. Indian J Pathol Microbiol.

[16] Aung TT, Chu A, Kondapi D, Mirchia K, Kaul P (2020). A case of pulmonary epithelioid hemangioendothelioma with literature review. Case Rep Oncol Med.

[17] Lau K, Massad M, Pollak C, Rubin C, Yeh J, Wang T (2011). Clinical patterns and outcome in epithelioid hemangioendothelioma with or without pulmonary involvement: insights from an internet registry in the study of a rare cancer. Chest.

[18] Errani C, Zhang L, Sung YS, Hajdu M, Singer S, Maki RG (2011). A novel WWTR1-CAMTA1 gene fusion is a consistent abnormality in epithelioid hemangioendothelioma of different anatomic sites. Genes Chromosomes Cancer.

[19] Huang H, Wang M, Zhu J (2024). Malignant pulmonary epithelioid hemangioendothelioma masquerading as lung adenocarcinoma: a possible radiological and pathological diagnostic pitfall. Pulmonology.

[20] Zheng Z, Wang H, Jiang H, Chen E, Zhang J, Xie X (2017). Apatinib for the treatment of pulmonary epithelioid hemangioendothelioma: a case report and literature review. Medicine (Baltim).

[21] Xue Z (2020). Progress in the diagnosis and treatment of pulmonary epithelioid endothelial tumors. Tumor Prev Treat.

[22] Rosenbaum E, Jadeja B, Xu B, Zhang L, Agaram NP, Travis W (2020). Prognostic stratification of clinical and molecular epithelioid hemangioendothelioma subsets. Mod Pathol.

[23] Bagan P, Hassan M, Le Pimpec Barthes F, Peyrard S, Souilamas R, Danel C (2006). Prognostic factors and surgical indications of pulmonary epithelioid hemangioendothelioma: a review of the literature. Ann Thorac Surg.

